# Effects of water deficit on fruit quality and water productivity of citrus under plastic film mulching in Western Hubei, China

**DOI:** 10.3389/fpls.2025.1498798

**Published:** 2025-04-03

**Authors:** Yun Zhong, Zhikun Huang, Kun Hao, Shijiang Zhu, Liangjun Fei, Jian Zeng, Zhiguang Dai, Yalin Wang

**Affiliations:** ^1^ Hubei Key Laboratory of Hydropower Engineering Construction and Management, China Three Gorges University, Yichang, China; ^2^ State Key Laboratory of Eco-hydraulics in Northwest Arid Region, Xi’an University of Technology, Xi’an, China; ^3^ Ganzhou Institute of Agricultural Sciences, Ganzhou, China; ^4^ College of Agricultural Equipment Engineering, Henan University of Science and Technology, Luoyang, China

**Keywords:** water deficit, film mulching, citrus yield, fruit quality, water use efficiency

## Abstract

**Introduction:**

The cultivation of Citrus sinensis Osbeck, the fruit with the largest planting scale and yield in Western Hubei Province of China, currently faces significant challenges related to low fruit quality and water use efficiency (WUE). This study aims to enhance citrus quality, yield, and WUE in the region by investigating the effects of water deficit and film mulching on 10-year-old citrus trees.

**Methods:**

From 2019 to 2021, three levels of water deficit (Light: 80%-90%, Moderate:70%-80%, Severe: 60%-70% of field capacity) and four mulching treatments (A: Japanese film, B: Dupont film, C: Chinese film, and no mulching) were implemented at the young fruit stage. Full irrigation (90%-100% of field capacity) was used as the control.

**Results and discussion:**

The light reflectance of films A, B, and C increased by 43.7%, 44.6%, and 6.3% respectively on sunny days compared to no mulching. Films A and B exhibited 2.2 times higher reflectivity than film C. Moderate water deficit - Japanese film (M-A) and moderate water deficit - Dupont film(M-B) treatments demonstrated the greatest improvement in citrus quality. Water deficit combined with film mulching resulted in an average increase in WUE of 10.90%-20.35% compared to full irrigation, and 8.96%-16.52% compared to no mulching. Mulching led to an average increase in citrus yield of 3.09%-16.48% compared to no mulching. The interaction between water deficit and film mulching significantly influenced both yield and WUE. From 2019-2021, M-A and M-B treatments yielded the highest citrus production, consistently demonstrating superior performance. Therefore, the better treatments would be a combination of M-A and M-B treatments, which correspond to soil moisture levels of 70% *θ_f_*–80% *θ_f_* during the young fruit period of citrus under mulching with films A and B. This combination was expected to enhance citrus quality, yield, and WUE. The outcome of this study may offer scientific basis and technical support for citrus irrigation management in Western Hubei, China.

## 1 Introduction

Citrus is the world’s popular fruit, which is rich in vitamin C, folate and dietary fiber, which can effectively prevent cancer and other diseases (Yu et al., 2024). Citrus planting is mainly distributed in tropical and subtropical areas between 30°N and 30°S, among which China has a long history of citrus planting, which is the origin and main production area of citrus (Dong et al., 2024; Poles et al., 2020). In 2022, China’s citrus planting area was nearly 2.99×10^6^ ha, and the output exceeded 4.46×10^7^ t, both ranking first in the world (Food and Agriculture Organization, 2022).

West Hubei is located in southwest China, mainly in mountainous and hilly areas. The agricultural planting structure of the region is mainly composed of characteristic economic crops such as citrus, tea and medicinal materials. Citrus is the fruit tree with the largest planting area and the most important economic status in Western Hubei region of China, and the citrus industry has become one of the pillar industries for rural economic development in the region, especially for farmers to leave poverty and become rich (Luo et al., 2025). However, although the region is one of the main producing areas of citrus, the overall quality of fruit is low, still needing to import from South Africa, Egypt, and Australia and other countries every year (Yi and Liu, 2022). Therefore, improving the fruit quality of citrus has become the core practical problem that the citrus industry in the region needs to be solved urgently.

Water plays an important regulatory role in the fruit quality of citrus (Lin et al., 2019). Citrus is a water-consuming tree species with poor drought tolerance, and it is very sensitive to water; scientific irrigation is the key technical measure for high quality and stable yield of citrus (Khan et al., 2022). In recent years, the problem of seasonal drought in Western Hubei region of China has become increasingly prominent (Wen and Chen, 2023), and the water resources for agricultural irrigation are obviously insufficient, while the local fruit farmers still continue use traditional flood irrigation methods, which not only causes low fruit quality and water-use efficiency (WUE) but also leads to local soil erosion and other problems (Hou et al., 2023). Therefore, to improve WUE and fruits quality, scholars have proposed a regulated deficit irrigation method that uses the physiological function of crops to save water (Arbizu-Milagro et al., 2023; Hao et al., 2022). Most studies have shown that reasonable water deficit could improve the quality of fruits without reducing yield or with very little reduction in yield, such as increasing excellent fruit percentage and soluble reducing sugar content (Abou Ali et al., 2024; Zhong et al., 2019; Saitta et al., 2021), fruit hardness increases slightly and fruit moisture content decreases slightly, making the fruit sweeter and easier to store (Zhong et al., 2019).

Film mulching technology has been promoted and applied in China with great success, and the mulching area and usage could continue to increase in the future, it is expected that the mulching area in China will expand to 23.4×10^6^ ha in 2025 (Qi et al., 2020). Therefore, film mulching technology could continue to grow and become irreplaceable in China’s agricultural production (Liu et al., 2024; Zhao et al., 2023). Film mulching affects fruit quality by changing soil temperature, water potential, and the light inside trees, significantly contributing to the yield and fruit quality of orchard (Pacheco et al., 2021; Zhao et al., 2022). Many scholars have confirmed that film mulching can induce the gene expression of sucrose synthase in citrus fruits, promote the synthesis of soluble sugar, and thus increase the sugar content of fruit, and significantly improve the soluble solid content, solid acid ratio and coloration of fruit (Jin et al., 2018; Duan et al., 2022; Yang et al., 2023). As a result, film mulching could significantly increase WUE and fruit quality, improve crop growth micro-environment (Song et al., 2023), which is especially suitable for citrus fruit trees with high requirements for soil environmental conditions, such as temperature and water.

Although extensive research has been conducted on regulated deficit irrigation and surface film mulching, these studies mostly focus on a single control factor such as different water deficit conditions or different film mulching conditions, and rarely analyze the interaction between water deficit and plastic film mulching. Therefore, this study tried to introduce surface film mulching technology on the basis of regulated deficit irrigation, and three common mulching films for experiment. By taking advantage of the water-retaining and water-controlling effect of mulching and its advantages in increasing fruit yield and improving fruit quality, the most suitable mulching film for regulating deficit irrigation was selected through in-depth research on the comprehensive effects of water deficit and mulching on citrus yield, quality and WUE, so as to solve the problems of soil water shortage and later yield reduction caused by water deficit. In order to provide theoretical basis and technical support for irrigation management, quality and efficiency improvement of citrus industry in western Hubei region of China.

## 2 Material and methods

### 2.1 Experimental location and materials

**Figure 1:**
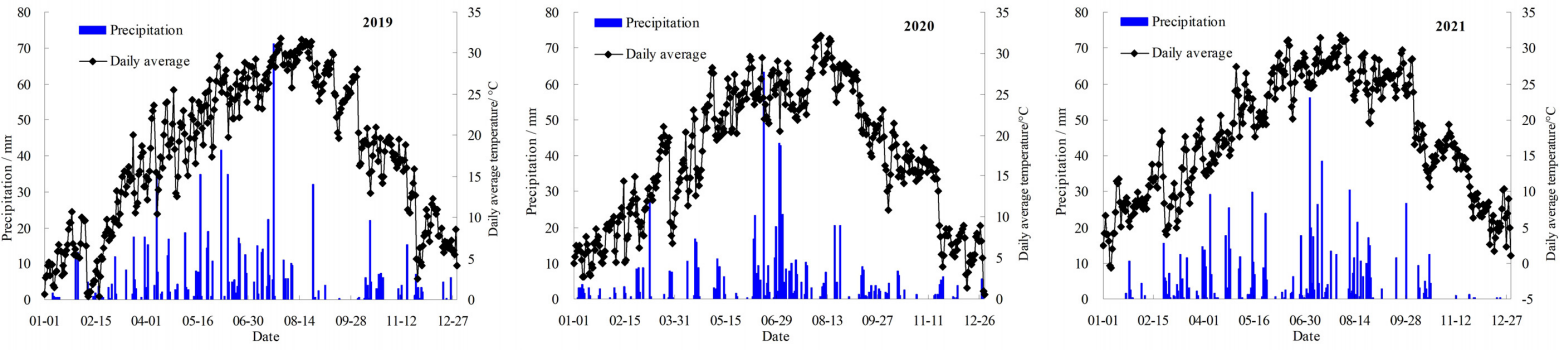
Daily precipitation and average temperature in 2019-2021.

**Figure 2:**
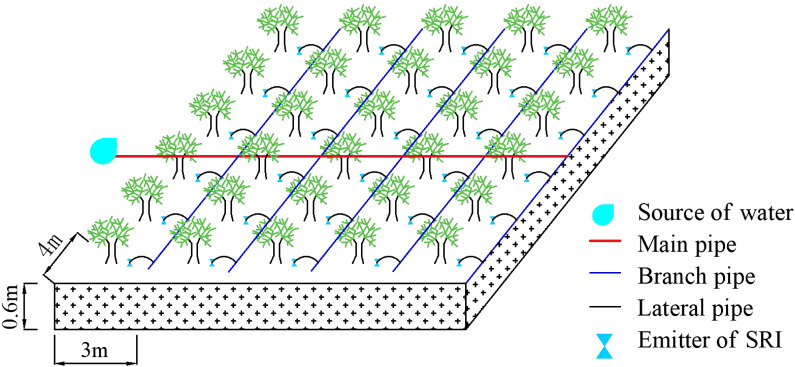
Sketch of test arrangement. SRI is surge root irrigation.

The experiment was carried out in Cangwubang Citrus Experimental orchard (30°75′ N, 110°41′ E, altitude of 343 m) of China Three Gorges University from March 2019 to October 2021. The orchard covers an area of 1.4 ha, the average temperatures from 2019 to 2021 were 17.8, 17.3, and 17.6 °C, respectively, and the annual effective rainfall were 862, 745, and 825 mm (**Figure 1**). The average annual wind speed of 1.4 m s^-1^, average annual atmospheric humidity of 75.6%, and average annual sunshine hours of 1619.6 hours. The region features a subtropical continental monsoon climate and a national citrus dominant industrial area. The “Red Navel Orange” was selected as the research object due to its favorable fruit quality (Zacarías-Garcia et al., 2023), wide market demand, and extensive cultivation scale in the local area (Yi and Liu, 2022), with trees aged 10 years serving as the basis for the study. The citrus variety was identified as one of the key promotion varieties in Hubei Province, and its plant spacing is 4 m × 3 m. The citrus trees were obtained using conventional grafting methods, with *Fructus aurantii* as the rootstock, the plant height was 230–250 cm, stem diameter was 7.5–8.9 cm, canopy width was 250–275 cm. The root drill method was used to measure the root system of citrus trees in the depth of 0–60 cm, which accounted for 85.1% of the total number of trees in the depth of 0–2 m. Therefore, the depth of the wetting layer was planned to be 60 cm, and the experiment layout is shown in **Figure 2**. The type of soil in the experimental field is clay soil (USDA Soil Classification System), with a texture composition of 25% sand, 36% silt, and 39% clay. The soil bulk density is 1.38 g cm^−3^,and the field capacity is 20.01% (mass water content). The whole growth period of “Red Navel Orange” was divided into five growth stages: I (budding and flowering stage, from mid-March to late April), II (young fruit stage, from early May to late May), III (fruit swelling stage, from early June to mid-September), IV (color-changing and sugar-increasing stage, from late September to late October), and V (dormancy stage, from November to next February).

Three kinds of film were selected in the experiment: A (moisture permeable film of outer white and inner black; provided by Tokuyama Plastics, Japan; specification of 100 m × 2 m; outer layer is rainproof and breathable, and inner layer is non-woven fabric; available for 3–5 years), B (DuPont Tewei strong film, provided by DuPont; door width of 1.524 m and thickness of 60 g/m^2^), and C (domestic silver black double-color film, provided by Jiangsu Mikeduo Agricultural Film Development Company; door width of 1.5 m and thickness of 0.02 mm). All film mulching treatments in the same year were uniformly applied and removed at the same time. In 2019, 2020, and 2021, film mulching was applied starting from the young fruit stage on May 2, May 4, and May 1, respectively. The film was removed after fruit harvesting on November 7, November 14, and October 29, respectively. Before mulching, the orchard ground was levelled, large stones and weeds were removed, a drainage ditch was dug around, and the whole terraces were covered. The film was laid tightly and close to the ground and fixed with stones and soil bags around, so that rainwater could flow out from the film surface along the drainage ditch.

### 2.2 Experimental design

Based on previous planting experiences, the results were determined that red navel orange can survive when the soil moisture content was between 60% *θ_f_* and 100% *θ_f_* (where *θ_f_* is field capacity). Thus, three different water deficit levels were set at stage II in this experiment, namely low (L), moderate (M), and severe (S); the control ranges of soil moisture content were 80% *θ_f_*–90% *θ_f_*, 70% *θ_f_*–80% *θ_f_*, and 60% *θ_f_*–70% *θ_f_*, respectively, and irrigation to the upper limit when the soil moisture content was below the lower limit. Four different surface coverage levels (film A, B, C and no mulching) were set up, and full irrigation (F-0: 90% *θ_f_*–100% *θ_f,_* the irrigation methods commonly used by local citrus growers) was used as control to design a total of 13 groups of experimental treatments. The specific test scheme is shown in **Table 1**. A completely randomized block design was used in the experiment, with 3 citrus trees in each plot and 3 repeats in each treatment. 1 m waterproof board was used for anti-seepage isolation between the plots. Isolated rows (two empty columns between treatments) were set up between each treatment in the trial, and standard agronomic practices, such as pruning, ring stripping, insecticide spraying, and weed control, were used in all treatments.

**Table 1:** Experiment scheme of regulated deficit irrigation for citrus under plastic film mulching.

Treatment		Upper and lower limit of irrigation at II stage(%)	Plastic film
Low deficiency at stage II	L-A	80*θ_f_* ~ 90*θ_f_*	A
	L-B	80*θ_f_* ~ 90*θ_f_*	B
	L-C	80*θ_f_* ~ 90*θ_f_*	C
	L-0	80*θ_f_* ~ 90*θ_f_*	No film covering
Moderate deficiency at stage II	M-A	70*θ_f_* ~ 80*θ_f_*	A
	M-B	70*θ_f_* ~ 80*θ_f_*	B
	M-C	70*θ_f_* ~ 80*θ_f_*	C
	M-0	70*θ_f_* ~ 80*θ_f_*	No film covering
Severe deficiency at stage II	S-A	60*θ_f_* ~ 70*θ_f_*	A
	S-B	60*θ_f_* ~ 70*θ_f_*	B
	S-C	60*θ_f_* ~ 70*θ_f_*	C
	S-0	60*θ_f_* ~ 70*θ_f_*	No film covering
Full irrigation	F-0	90*θ_f_* ~ 100*θ_f_*	No film covering

*θ_f_* is field capacity; L, M, and S are low, moderate, and severe water deficit, respectively; A, B, and C are Japanese film, Dupont film, and Chinese film, respectively. Irrigation to the upper limit when the soil moisture content was below the lower limit.

**Figure 3:**
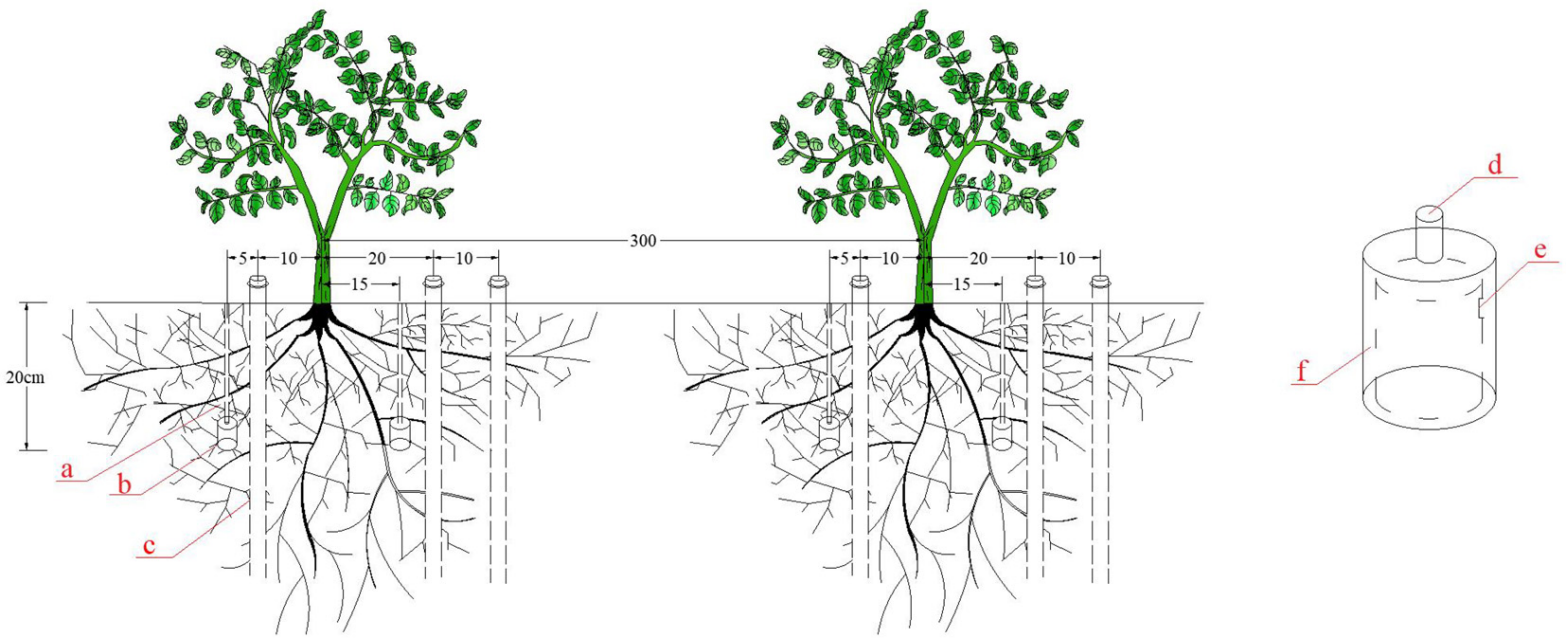
Surge root irrigation. (a) Microtube; (b) Emitter of SRI; (c) Trime tube; (d) Water intake; (e) Water outlet; (f) Labyrinth channel. Unit is cm.

In accordance with the local fertilization method, citrus was fertilized twice during the whole reproductive period, in phase I (base fertilizer), and in phase III (follow-up fertilizer). The amount and method of fertilization were the same in all treatments, in which the amount of organic fertilizer (cow dung) was 8.0 kg plant^-1^ in the whole growth period, and the ratio of two times was 3:2, which was added by hole application at 30 cm from the trunk of fruit trees. The amounts of potassium dihydrogen phosphate (including P_2_O_5_ 52% and K_2_O 34%) and potassium sulfate (including K_2_O 52%) in the whole growth period were 0.1 and 0.2 kg plant^-1^, respectively, and the ratio of the two applications was 3:2. Nitrogenous fertilizer (urea with N content of 46%) was applied at the rate of 0.25 kg plant^-1^ in the whole growth period, and the ratio of two times was 2:1. Nitrogenous, phosphate, and potassium fertilizers were applied to soil by using Venturi fertilizer applicator and pouring surge root irrigation. In accordance with the results of the previous soil profile irrigation test in the test area, a surge root irrigation emitter (PVC material) was arranged at 15 cm on the east and west sides of the trunk of citrus trees and a buried depth of 20 cm. The flow rate of the emitter at the time of irrigation was 3 L h^-1^, the outer diameter was 4 cm, the flow index was about 0.5, and it was in a turbulent state. The difference between the working pressure of the emitter at the beginning and end of the same pipe was less than 2%, the pressure change was small, and the difference in the flow rate of the emitter was less than 5%, so the uniformity of the outflow of each emitter was high (**Figure 3**).

### 2.3 Main observation indices and methods

#### 2.3.1 Meteorological data

These data were obtained through a fully automatic weather station at the test site, mainly including temperature, air relative humidity, atmospheric pressure, solar radiation intensity, wind speed, wind direction, and rainfall. The measurements were taken every 1 min and recorded every 30 min.

#### 2.3.2 Surface reflectivity of citrus orchards

After plastic film mulching, the reflectance of 11 treatments was measured by CIRAS-2 photosynthesis system under sunny and cloudy weather conditions, with three trees per treatment for three replicates. The measurement time was between 11:00 and 13:00, and the light intensity of each tree was measured at four points 1 m away from the main trunk in the east, south, west, and north. The incident light intensity was measured at the ground surface. The reflected light intensity at different heights was measured at the vertical heights of 0.8 and 1.4 m at each point, and the reflectivity was calculated as follows: reflectance (%) = reflected light/incident light × 100%. The light intensity unit is μmol m^-2^ s^-1^.

#### 2.3.3 Soil moisture content

Soil moisture content was measured using a calibrated TRIME-T3 tubular TDR system with TRIME tubes buried at horizontal distances of 10, 20, and 30 cm from the irrigator, as shown in **Figure 3**. It was measured once every 3 days at every 10 cm-deep soil layer until it reached a planned wetting layer depth of 60 cm, and the average value was taken to determine the irrigation volume. Each treatment was repeated three times. When the soil moisture content was found to be lower than the lower limit, irrigation was performed; otherwise, no irrigation was performed.

#### 2.3.4 Irrigation amount

Equation 1 is the calculation method of irrigation amount.


(1) $$I=0.1\;\gamma \cdot z\cdot p\cdot S(\theta_{\max}-\theta_{\min})/\eta$$

where *I* is the amount of irrigation, L; *γ* is the soil bulk density, g cm^-3^; *z* is the planned wetting layer depth, m; *p* is the wetting ratio; *S* is the single tree area, m^2^; *θ*_max_ and *θ*_min_ are the upper and lower limits of the soil moisture content (percentage of soil mass), respectively; and *η* is the utilization coefficient of irrigation water.

#### 2.3.5 Fruit quality

(i) Fruit external quality index: during fruit picking (November 7, 2019, November 14, 2020, and October 29, 2021), two fruits were selected in each of the four directions of the citrus tree. A total of eight fruits were selected for each tree, using the accuracy of 0.01 mm vernier calipers to measure the vertical and horizontal diameter of the fruit (*r, h*) and peel thickness. The transverse diameter measures the upper, middle, and lower parts of the fruit to obtain the average transverse diameter. The ratio of the longitudinal diameter to the transverse diameter is the fruit shape index. The single fruit weight of each citrus tree was measured by an electronic scale with an accuracy of 0.01 g, and the sum of single fruit weight was the yield per plant of citrus. The peel color was measured by CR-400 colorimeter. The red-green difference a* was measured on the colorimeter. Before the measurement, the standard whiteboard was used for correction. Each fruit surface was measured four times at different positions, and the measured data were the relative values of the whiteboard. The citrus fruits were graded in accordance with the grades and specifications of citrus fruits (Agricultural Industry Standard of China NY/T 1190-2006). The special and first grade fruits were defined as superior fruits, and the excellent fruit percentage of citrus was calculated.

(ii) Fruit internal quality index: after the external quality index of fruit was measuring, the fruit was cleaned and wiped dry with distilled water. Half of the juice was taken by dichotomy, and the juice yield was determined by pressing method. The other half was ground and mixed with a mixer to determine the vitamin C content, soluble solid content, soluble reducing sugar content, and titratable acid content by using 2,6-dichloro indophenol reagent (Sun et al., 2022), PR-32α handheld sugar meter (Zhong et al., 2019), thermal titration with Fehling reagent (Zhong et al., 2019), and NaOH titration (Hao et al., 2022), respectively. Each treatment was repeated three times.

#### 2.3.6 Water use efficiency

Equation 2 is the calculation method of citrus water consumption (*ET*, mm).


(2) $$ET=\Delta W+I+P_{r}+G-D-R$$

Where Δ*W* is the reduction of soil water storage at two measurement intervals, mm; *I* is the irrigation amount, mm; *P_r_* is the effective rainfall, mm; *G* is the groundwater recharge, mm; *D* is the deep leakage, mm; *R* is the surface runoff, mm.

The mulching method used in the experiment was full ground cover, with plastic film covering the entire terrace, so the rainfall was not taken into account. The groundwater at the experimental location was at more than 20 m below the ground level, so the groundwater recharge was not considered. The experimental irrigation method being surge-root irrigation, with a small flow rate and low irrigation quota, so the deep leakage and surface runoff caused by irrigation can be ignored. Thus, Equation 2 could thus be simplified to Equation 3.


(3) ET=ΔW+I

Equation 4 is the calculation method of water use efficiency (*WUE*, kg m^-3^) is as follows:


(4) WUE=0.1×Y/ET

where *Y* is yield, kg ha^-1^.

### 2.4 Data analysis

Statistical analysis of the data was performed using Excel (v. 2013, Microsoft Corp., USA), and variance analysis was conducted using SPSS statistical software (v. 21.0, SPSS Inc., 2013). The significance of the treatment effect was determined using the F-test, and means were compared using the least significant difference (LSD) at the 5% level of significance.

## 3 Results

### 3.1 Effects of water deficit on light intensity in the middle and lower canopies of citrus under plastic film mulching

**Figure 4:**
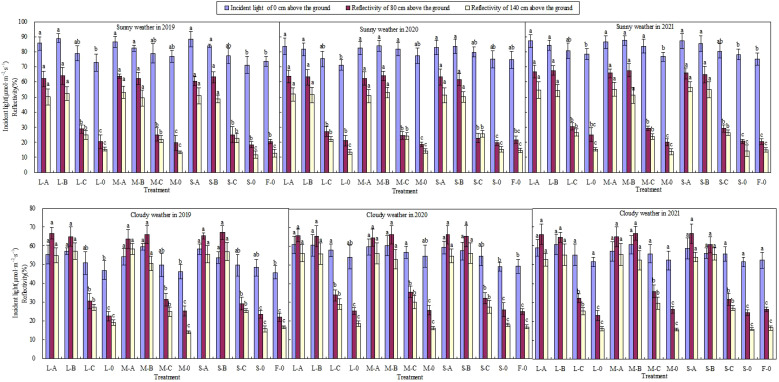
Light reflectivity of middle and lower canopy of citrus with different treatments. L, M, and S are low, moderate, and severe water deficit, respectively; A, B, and C are Japanese film, Dupont film, and Chinese film, respectively. Different small letters indicate values that are significantly different at the P < 0.05 level for comparisons within same year. Data shown are means ± standard error of the means (*n*=3). Error bars represent standard error.

Water deficit at stage II had no significant effect on the light reflectance of the middle and lower canopies of citrus, while films A, B, and C had significantly increase the light reflectance (**Figure 4**). At the vertical height of 80 cm above the ground, the light reflectance of films A, B, and C measured on sunny days increased by 43.7%, 44.6%, and 6.3%, respectively, compared to the treatment without film mulching. The reflectance at 140 cm decreased compared to 80 cm, and the light reflectance of the three types of film increased by 39.9%, 38.2%, and 5.6%, respectively. The reflectance of films A and B were about 2.2 times that of film C, and the difference reached an extremely significant level (*P* < 0.01). The reflectance of different film measured in cloudy days was slightly higher than in sunny days, films A and B showed more significant reflectance than film C and no film, with extremely significant differences (*P* < 0.01). Meanwhile, the differences between film C and no film reached significant levels (*P* < 0.05). The reflectance of films A, B, and C increased by 41.8%, 40.8%, and 9.6% at a vertical height of 80 cm compared with that of no film, respectively. The interaction between water deficit and plastic film had no significant effect on the light reflectance of canopy (**Table 2**).

**Table 2:** Significance analysis of various parameters of citrus under different treatments.

Factors	Significance test (F value)													
	Reflectivity	Fruit shape index	Single fruit weight	Coloring degree	Pericarp thickness	Excellent fruit percentage	Juice yield	Soluble solids content	Soluble reducing sugar	Titratable acid	Vitamin C	Yield	ET	WUE
Water deficit	0.63	0.72	7.64 **	0.53	0.57	15.42 **	8.41 *	2.81 *	4.36 *	0.80	0.21	3.92 *	18.01 **	7.49 **
Plastic film	40.58 **	0.34	3.67 *	51.02 **	2.38 *	10.67 **	0.66	5.42 **	1.65 *	6.13 *	3.58 *	4.14 *	2.14 *	4.67 *
Water deficit×Plastic film	0.92	0.50	2.79 *	1.72 *	0.21	3.10 *	1.22 *	1.44 *	1.31 *	0.30	0.12	1.58 *	1.92 *	1.70 *

*mean significant difference (P<0.05) and **mean extremely significant difference (P<0.01).

### 3.2 Effects of water deficit on quality of citrus under plastic film mulching

The fruit shape index was not significantly affected by film mulching and water deficit, which was relatively stable (**Table 3**). These two factors had a greater effect on single fruit quality, and the interaction between the two had a significant effect on single fruit quality (*P* < 0.05). Low deficiency at stage II significantly increased single fruit weight (*P* < 0.05), moderate deficiency had no significant effect on single fruit weight, whereas severe deficiency significantly reduced single fruit weight (*P* < 0.05). Under the same water deficit condition, the effect of film mulching on single fruit weight also reached a significant level (*P* < 0.05). Taking the moderate deficiency in 2021 as an example, the single fruit weight of M-A, M-B, and M-C treatments increased by 48.77%, 45.21%, and 28.78%, respectively, compared with that of M-0 treatment. The effect of water deficit at stage II on coloring degree was not significant (*P* > 0.05), whereas the three kinds of film mulching could significantly improve the fruit coloring degree (*P* < 0.05). From 2019 to 2021, the fruit coloring degree of films A, B, and C increased by 80.25%, 76.43%, and 30.32%, respectively, compared with that of the non-mulching treatment. The effect of films A and B was significant, and the difference was significant compared with that of film C and non-mulching (*P* < 0.05). Meanwhile, the difference between film C and non-mulching was also significant (*P* < 0.05). In 2019, the lighting conditions were more favorable, resulting in an overall higher fruit coloring degree compared to 2020 and 2021. For instance, under full irrigation, the coloring degree of the F-0 treatment in 2019 was 10.76% and 15.89% higher than that in 2020 and 2021, respectively. Water deficit treatment at stage II had slight effect on pericarp thickness, whereas the different film mulching had great effect on pericarp thickness. Three kinds of film mulching could significantly reduce pericarp thickness. Moderate deficiency and film mulching significantly increased the excellent fruit rate, among which low deficiency significantly increased the excellent fruit percentage (*P* < 0.05), moderate deficiency had no significant effect on the excellent fruit percentage, and severe deficiency significantly decreased the excellent fruit percentage (*P* < 0.05). The interaction between water deficit and plastic film also had a significant effect on the excellent fruit percentage (**Table 2**).

**Table 3:** Effects of water deficit on the appearance quality of citrus at stage II under film mulching conditions.

Treatment		2019					2020					2021				
		Fruit shape index	Single fruit weight (g)	Coloring degree	Pericarp thickness(cm)	Excellent fruit percentage (%)	Fruit shape index	Single fruit weight (g)	Coloring degree	Pericarp thickness(cm)	Excellent fruit percentage(%)	fruit shape index	Single fruit weight (g)	Coloring degree	Pericarp thickness(cm)	Excellent fruit percentage(%)
Low deficiency	L-A	0.89 ± 0.12b	201.22 ± 22.37b	25.23 ± 4.71ab	0.39 ± 0.04bc	73.14 ± 6.89ab	0.92 ± 0.11a	217.53 ± 34.18ab	28.09 ± 2.73a	0.41 ± 0.02b	74.85 ± 4.18b	0.89 ± 0.13a	207.01 ± 32.23ab	24.82 ± 4.58ab	0.38 ± 0.01c	77.57 ± 7.20ab
	L-B	0.93 ± 0.03a	214.82 ± 35.12ab	29.10 ± 3.08a	0.37 ± 0.03c	73.27 ± 8.63ab	0.90 ± 0.08ab	208.43 ± 21.02b	24.30 ± 4.52ab	0.39 ± 0.03bc	77.71 ± 6.72ab	0.91 ± 0.10a	205.49 ± 34.59ab	23.70 ± 4.03ab	0.37 ± 0.02c	74.57 ± 3.57b
	L-C	0.91 ± 0.05ab	189.37 ± 21.09c	22.76 ± 2.42b	0.42 ± 0.02b	68.74 ± 5.52b	0.95 ± 0.15a	194.52 ± 31.35bc	20.27 ± 1.92b	0.44 ± 0.04ab	67.85 ± 5.01c	0.94 ± 0.08a	192.76 ± 21.41b	20.21 ± 2.13b	0.43 ± 0.04ab	70.43 ± 8.86bc
	L-0	0.90 ± 0.11ab	178.42 ± 31.26cd	19.13 ± 4.07bc	0.45 ± 0.02a	62.71 ± 4.21c	0.93 ± 0.13a	185.97 ± 19.75c	18.18 ± 4.34bc	0.47 ± 0.02a	63.57 ± 7.73cd	0.89 ± 0.14a	173.92 ± 18.96c	16.39 ± 1.79c	0.45 ± 0.03a	66.71 ± 4.30c
Moderate deficiency	M-A	0.87 ± 0.07b	225.43 ± 27.31a	28.02 ± 2.68a	0.37 ± 0.01c	79.11 ± 8.06a	0.88 ± 0.11b	231.27 ± 25.21a	25.05 ± 4.01ab	0.36 ± 0.03c	80.42 ± 6.87a	0.93 ± 0.07a	223.71 ± 23.72a	27.78 ± 2.35a	0.37 ± 0.02c	82.96 ± 7.38a
	M-B	0.95 ± 0.11a	229.27 ± 23.57a	29.65 ± 2.07a	0.40 ± 0.03bc	77.29 ± 6.23a	0.92 ± 0.03a	227.74 ± 18.92a	28.74 ± 2.28a	0.37 ± 0.03c	81.28 ± 9.17a	0.91 ± 0.09a	218.35 ± 17.76a	27.52 ± 3.10a	0.40 ± 0.04bc	81.57 ± 8.24a
	M-C	0.91 ± 0.13ab	204.63 ± 17.24b	23.58 ± 1.82b	0.43 ± 0.01b	69.29 ± 4.89b	0.87 ± 0.08b	189.43 ± 23.53c	21.17 ± 1.72b	0.42 ± 0.02b	73.28 ± 4.27b	0.92 ± 0.13a	193.64 ± 21.55b	20.56 ± 1.82b	0.42 ± 0.02b	67.14 ± 5.22c
	M-0	0.88 ± 0.12b	166.19 ± 26.63d	18.92 ± 4.26bc	0.44 ± 0.03ab	53.14 ± 4.01de	0.93 ± 0.14a	179.02 ± 35.22cd	16.67 ± 1.90c	0.45 ± 0.01a	59.42 ± 3.85d	0.88 ± 0.14a	150.37 ± 31.94de	18.41 ± 3.41bc	0.46 ± 0.01a	63.42 ± 7.55cd
Severe deficiency	S-A	0.95 ± 0.10a	193.71 ± 33.56bc	28.33 ± 2.21a	0.37 ± 0.02c	62.86 ± 6.51c	0.92 ± 0.11a	179.25 ± 33.19cd	24.59 ± 4.69ab	0.36 ± 0.03c	70.85 ± 8.74bc	0.95 ± 0.08a	175.27 ± 17.86c	28.30 ± 2.16a	0.37 ± 0.02c	64.07 ± 6.27cd
	S-B	0.92 ± 0.08a	179.08 ± 34.04cd	27.90 ± 1.62a	0.38 ± 0.01c	58.14 ± 8.47cd	0.95 ± 0.12a	180.02 ± 36.75cd	27.50 ± 3.23a	0.39 ± 0.04bc	63.85 ± 6.54cd	0.94 ± 0.05a	164.86 ± 34.73cd	27.65 ± 1.66a	0.39 ± 0.03bc	64.11 ± 8.70cd
	S-C	0.93 ± 0.09a	172.29 ± 29.17cd	21.68 ± 3.02b	0.43 ± 0.02b	51.43 ± 8.25de	0.89 ± 0.15ab	169.89 ± 21.45d	20.76 ± 3.10b	0.42 ± 0.02b	64.29 ± 9.30cd	0.90 ± 0.07a	150.25 ± 32.17de	23.57 ± 3.08ab	0.41 ± 0.01b	60.75 ± 4.38d
	S-0	0.89 ± 0.11b	151.52 ± 20.14e	19.34 ± 4.39bc	0.44 ± 0.04ab	48.29 ± 4.32e	0.87 ± 0.09b	160.29 ± 16.84e	17.32 ± 4.82bc	0.45 ± 0.01a	52.31 ± 3.53e	0.88 ± 0.13a	141.21 ± 13.52e	15.71 ± 1.20c	0.45 ± 0.01a	50.41 ± 3.24e
Full irrigation	F-0	0.87 ± 0.15b	167.30 ± 22.86d	17.51 ± 3.18c	0.46 ± 0.01a	55.71 ± 5.49d	0.87 ± 0.09b	170.24 ± 15.68d	15.81 ± 1.47c	0.47 ± 0.02a	59.75 ± 4.02d	0.89 ± 0.11a	158.95 ± 19.47d	15.14 ± 1.45c	0.46 ± 0.02a	60.80 ± 5.10d

L, M, and S are low, moderate, and severe water deficit, respectively; A, B, and C are Japanese film, Dupont film, and Chinese film, respectively. For each year, values within a column followed by a different letter are significantly different at P < 0.05 according to an LSD test.

Water deficit at stage II had slight effect on titratable acid and vitamin C content and significant effect on juice yield, soluble solids, and soluble reducing sugar content (**Table 4**). With the increase in water deficit, the juice yield decreased significantly. Compared with full irrigation, the juice yield of low deficit treatment decreased by 12.94% to 4.06%, moderate deficit treatment decreased by 18.09% to 11.44%, and severe deficit treatment decreased by 23.74% to 19.33% from 2019 to 2021. Soluble solids and soluble reducing sugar content increased significantly. Under the same water deficit condition, film mulching had minimal effect on juice yield, but it significantly increased the vitamin C content, soluble solids, and soluble reducing sugar content and decreased the titratable acid content (*P* < 0.05). Among them, the films A and B had the best effect, and the difference was significant compared with that of film C and no mulching (*P* < 0.05). Taking moderate deficiency as an example, the vitamin C contents, soluble solids, and reducing sugar content in the two kinds of film mulching were 46.05%–25.11%, 14.01%–9.57%, and 28.07%–16.77% higher than those of no mulching, respectively. The interaction between water deficit and film mulching had significant effects on juice yield, soluble solids, and soluble reducing sugar content (**Table 2**).

**Table 4:** Effects of water deficit on the intrinsic quality of citrus at stage II under film mulching conditions.

Treatment		2019					2020					2021				
		Juice yield (%)	Soluble solids content (%)	Soluble reducing sugar (%)	Titratable acid (%)	Vitamin C (mg 100mL^-1^)	Juice yield (%)	Soluble solids content (%)	Soluble reducing sugar (%)	Titratable acid (%)	Vitamin C (mg 100mL^-1^)	Juice yield (%)	Soluble solids content (%)	Soluble reducing sugar (%)	Titratable acid (%)	Vitamin C (mg 100mL^-1^)
Low deficiency	L-A	50.33 ± 3.05ab	13.35 ± 1.05bc	3.16 ± 0.23c	0.65 ± 0.07c	30.61 ± 3.48a	51.24 ± 2.45b	13.09 ± 0.56b	3.58 ± 0.36bc	0.69 ± 0.08c	31.68 ± 2.32a	51.97 ± 2.03b	13.72 ± 0.65bc	3.68 ± 0.22b	0.66 ± 0.05c	31.25 ± 2.12a
	L-B	47.88 ± 2.11b	12.92 ± 0.52c	3.23 ± 0.26c	0.63 ± 0.10c	32.51 ± 4.20a	50.64 ± 3.02b	13.26 ± 0.35b	3.39 ± 0.17c	0.66 ± 0.04c	29.78 ± 2.50a	51.82 ± 2.10b	13.35 ± 0.31c	3.75 ± 0.14b	0.63 ± 0.08c	31.62 ± 2.66a
	L-C	46.94 ± 1.07b	12.14 ± 0.36d	2.87 ± 0.18d	0.78 ± 0.07b	26.85 ± 2.03b	48.78 ± 1.27b	12.43 ± 0.21c	3.05 ± 0.18d	0.76 ± 0.07b	27.21 ± 1.82b	50.27 ± 1.33b	12.51 ± 0.33d	3.45 ± 0.20c	0.76 ± 0.05b	23.70 ± 3.93bc
	L-0	45.67 ± 2.33bc	11.79 ± 0.82de	2.69 ± 0.37de	0.86 ± 0.09a	23.56 ± 2.33c	48.31 ± 1.02b	12.07 ± 1.02cd	2.74 ± 0.20e	0.89 ± 0.10a	25.76 ± 3.58bc	48.33 ± 3.37bc	12.03 ± 0.72de	3.21 ± 0.36cd	0.86 ± 0.10a	24.78 ± 3.50bc
Moderate deficiency	M-A	44.11 ± 1.25c	14.05 ± 0.91ab	3.63 ± 0.21b	0.63 ± 0.11c	28.89 ± 4.06ab	47.01 ± 3.41bc	13.58 ± 1.11ab	3.76 ± 0.25b	0.61 ± 0.10c	31.51 ± 2.20a	48.27 ± 3.02bc	14.32 ± 0.86ab	3.87 ± 0.39ab	0.63 ± 0.08c	29.67 ± 2.36a
	M-B	44.47 ± 2.01c	13.62 ± 0.82b	3.65 ± 0.11b	0.68 ± 0.06c	31.17 ± 3.71a	46.25 ± 1.31c	13.51 ± 1.07ab	3.87 ± 0.36ab	0.62 ± 0.07c	32.13 ± 1.94a	48.39 ± 2.67bc	14.03 ± 0.25b	3.83 ± 0.30ab	0.68 ± 0.05c	31.40 ± 2.62a
	M-C	43.25 ± 0.97c	12.91 ± 0.46c	3.21 ± 0.19c	0.75 ± 0.05b	26.91 ± 2.22b	45.30 ± 2.11c	12.39 ± 0.46c	3.21 ± 0.37cd	0.78 ± 0.06b	26.03 ± 3.32bc	46.02 ± 2.73c	13.32 ± 0.59c	3.41 ± 0.18c	0.78 ± 0.07b	26.35 ± 1.66b
	M-0	42.97 ± 3.08cd	12.43 ± 1.13cd	2.85 ± 0.13d	0.87 ± 0.08a	22.26 ± 2.81c	44.97 ± 1.37c	12.02 ± 0.85cd	3.09 ± 0.26d	0.86 ± 0.05a	22.68 ± 2.27c	45.87 ± 2.89c	12.56 ± 0.38d	3.28 ± 0.34cd	0.84 ± 0.08a	21.48 ± 1.38c
Severe deficiency	S-A	41.90 ± 1.05d	14.75 ± 0.89a	4.03 ± 0.20a	0.63 ± 0.11c	30.52 ± 3.58a	43.54 ± 2.84cd	13.89 ± 0.47a	4.02 ± 0.28a	0.61 ± 0.09c	30.26 ± 1.69a	42.32 ± 2.47d	14.62 ± 0.47a	4.09 ± 0.24a	0.63 ± 0.11c	30.24 ± 2.32a
	S-B	41.72 ± 1.78d	14.51 ± 0.48a	3.96 ± 0.27a	0.66 ± 0.09c	30.81 ± 2.83a	43.39 ± 2.61cd	14.01 ± 0.52a	4.16 ± 0.30a	0.66 ± 0.05c	29.61 ± 1.46a	44.08 ± 3.51cd	14.73 ± 0.52a	4.13 ± 0.12a	0.65 ± 0.09c	30.72 ± 2.03a
	S-C	41.05 ± 2.02d	13.64 ± 0.65b	3.67 ± 0.26b	0.77 ± 0.05b	26.07 ± 2.52b	42.12 ± 1.07d	13.14 ± 0.41b	3.72 ± 0.19b	0.78 ± 0.06b	27.53 ± 2.01b	42.02 ± 2.73d	14.01 ± 0.37b	3.72 ± 0.27b	0.76 ± 0.05b	26.83 ± 2.16b
	S-0	40.38 ± 2.33d	13.31 ± 1.06bc	3.43 ± 0.38bc	0.83 ± 0.14ab	23.14 ± 2.80c	42.01 ± 1.34d	12.32 ± 0.68c	3.37 ± 0.21c	0.81 ± 0.12ab	23.15 ± 2.34c	41.67 ± 3.05d	13.02 ± 0.82cd	3.61 ± 0.38bc	0.84 ± 0.09a	23.46 ± 3.18bc
Full irrigation	F-0	52.46 ± 2.27a	11.38 ± 0.63e	2.54 ± 0.25e	0.87 ± 0.07a	22.91 ± 3.07c	54.72 ± 3.22a	11.41 ± 0.71d	2.71 ± 0.28e	0.89 ± 0.10a	25.09 ± 3.42bc	54.64 ± 2.01a	11.67 ± 0.35e	3.05 ± 0.25d	0.87 ± 0.11a	24.04 ± 2.48bc

L, M, and S are low, moderate, and severe water deficit, respectively; A, B, and C are Japanese film, Dupont film, and Chinese film, respectively. For each year, values within a column followed by a different letter are significantly different at P < 0.05 according to an LSD test.

### 3.3 Comprehensive evaluation of fruit quality

Principal component analysis was performed on 10 citrus quality indicators of 13 treatments. The initial data of each quality index were standardized and converted into dimensionless data with a mean value of 0 and a standard deviation of 1 to eliminate the influence of different units and data dimensions. After each index was standardized, the principal component score was calculated in accordance with the result and the factor load matrix (Gizaw et al., 2016). The formulas are Equations 5 and 6.


(5) $$\begin{array}{c} F_{1}=1.014X_{1}+1.308X_{2}+1.826X_{3}-1.823X_{4}+1.288X_{5}\\ -0.381X_{6}+1.630X_{7}+1.505X_{8}-1.859X_{9}+1.781X_{10} \end{array}$$

(6) $$\begin{array}{c} F_{2}=-0.405X_{1}+1.349X_{2}-0.052X_{3}-0.002X_{4}+1.436X_{5}\\ +1.861X_{6}-0.987X_{7}-1.049X_{8}-0.070X_{9}+0.368X_{10} \end{array}$$

Where *F*_1_ and *F*_2_ are the scores of the first and second principal components, respectively; *X*_1_, *X*_2_,…, *X*_10_ are the 10 measured quality indicators of citrus.

The obtained two principal components (*F*_1_ and *F*_2_) and the ratio of the eigenvalues corresponding to each principal component (*F*_1_: 0.62, *F*_2_: 0.20) to the cumulative eigenvalues of the extracted principal components (0.82) were used as weights to calculate the principal component synthesis model (Equation 7):


(7) $$F=(0.62/0.82)F_{1}+(0.20/0.82)F_{2}$$

**Figure 5:**
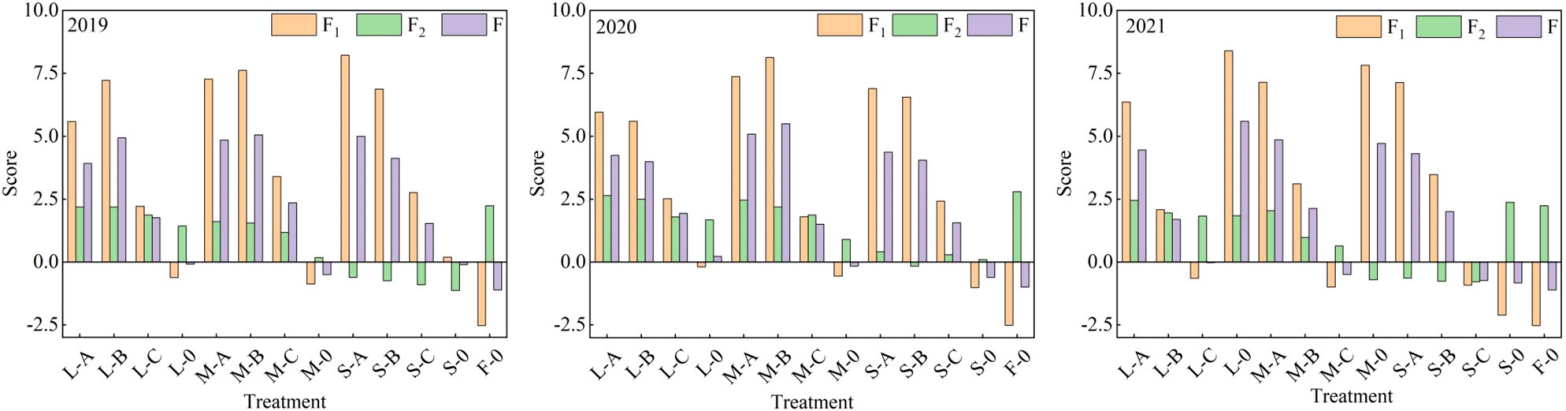
Principal component score and comprehensive score of citrus under different treatment modes. L, M, and S are low, moderate, and severe water deficit, respectively; A, B, and C are Japanese film, Dupont film, and Chinese film, respectively. *F*_1_ and *F*_2_ are the scores of the first and second principal components, respectivel. *F* is comprehensive score.

The comprehensive scores of each treatment mode in different years were calculated (**Figure 5**). The higher the comprehensive score, the better the adaptability of the selected film under the deficit treatment mode and the better the comprehensive quality. The results show that the M-B treatment ranked first in comprehensive scores in both 2019 and 2020, while the M-A treatment ranked first in 2021. The fruit shape index and single fruit weight of M-B treatment were the best in 2019, and the excellent fruit percentage and vitamin C content of M-B treatment were the best in 2020. The single fruit weight and excellent fruit percentage of M-A treatment were the best in 2021. Among the treatments ranked first in each year, the proportion of moderate deficiency treatment was 100%, indicating that moderate deficiency treatment was the best treatment to improve citrus quality, and the most suitable film were films A and B. The lowest score was found in F-0 treatment, indicating that sufficient irrigation could not improve the quality of citrus, and citrus under film mulching could improve the quality of citrus.

### 3.4 Effects of water deficit on yield and WUE of citrus under plastic film mulching

**Figure 6:**
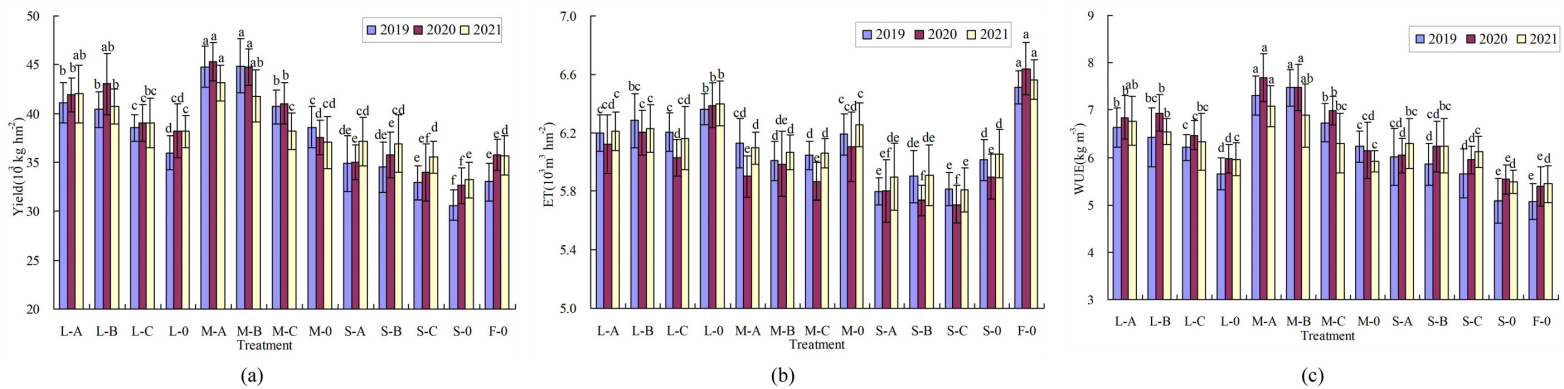
Effects of water deficit on the yield, ET (Water consumption) and WUE (Water use efficiency) of citrus at young fruit stage under film mulching conditions. L, M, and S are low, moderate, and severe water deficit, respectively; (a-c) are Japanese film, Dupont film, and Chinese film, respectively. F-0 is full irrigation and no film covering. Different small letters (a, b, c, etc) indicate values that are significantly different at the P < 0.05 level for comparisons within same year. Data shown are means ± standard error of the means (n=3). Error bars represent standard error.

Citrus yield was significantly affected by water deficit (**Figure 6a**). Comparing the three treatments of L-0, M-0, and S-0 with F-0 treatment showed that low and moderate-deficiency at stage II significantly increased citrus yield, whereas severe deficiency significantly reduced it. Under the same water deficit condition, the effect of film mulching on citrus yield also reached a significant level (P < 0.05). Taking 3 years of severe deficiency as an example, the citrus yield of the three film mulching treatments (S-A, S-B, and S-C) increased on average by 10.95%, 11.11%, and 6.19%, respectively, compared with that of the non-film mulching treatment (S-0). The interaction between water deficit and film mulching had a significant effect on citrus yield (**Table 2**). The citrus yield without film mulching (S-0) was significantly reduced by 7.20% (P < 0.05) compared with F-0 treatment, whereas the yield of three treatments (S-A, S-B, and S-C) under film mulching was not significantly different from that of F-0 treatment (P > 0.05), indicating that film mulching could effectively improve citrus yield and weaken the adverse effect of severe water deficit on yield.

The calculated irrigation amount of each treatment is shown in **Table 5**. The water consumption (ET) of each treatment decreased significantly with the increase of water deficit degree (**Figure 6b**). Taking no film mulching as an example, the ET of L-0, M-0, and S-0 treatments decreased by 3.75%–2.32%, 8.04%–4.73%, and 11.13%–7.65%, respectively, compared with that of F-0 treatment from 2019 to 2021. Under the same water deficit condition, the three kinds of film could significantly reduce the ET of citrus trees (P < 0.05). In 2019–2021, the ET of films A, B, and C decreased by 4.14%–0.99%, 3.00%–1.21%, and 5.63%–2.35%, respectively, compared with that of no mulching, indicating that the water-saving effect of film C was better than that of films A and B. The overall ET of citrus trees in 2020 was lower than in the other two years, due to the combined effects of climatic conditions such as rainfall and sunlight intensity. The WUE of citrus trees in each treatment was higher than that in the control group with the largest water consumption (F-0) (**Figure 6c**). Water deficit and film mulching could effectively improve the WUE of citrus trees, and the interaction between the two also had a significant effect on WUE (**Table 2**). The WUE of M-A and M-B treatments was the highest, which was greater than 7.00 kg m^-3^ (except for M-B treatment in 2021); the WUE of the two treatments increased by 44.17% and 47.34%, respectively, compared with that of F-0 treatment in 2019. In 2020, the WUE of M-A and M-B treatments increased by 42.61% and 38.73%, respectively, and in 2021, it increased by 30.11% and 26.66%, respectively.

**Table 5:** Irrigation amount of each treatment of citrus in 2019-2021.

Treatment		Irrigation amount (m^3^ ha^-1^)		
		2019	2020	2021
Low deficiency	L-A	342.7	347.7	349.3
	L-B	353.0	346.9	344.8
	L-C	347.7	332.1	342.6
	L-0	363.9	365.8	369.6
Moderate deficiency	M-A	337.1	330.1	343.2
	M-B	332.6	335.3	332.7
	M-C	335.1	323.4	338.2
	M-0	353.7	354.4	357.3
Severe deficiency	S-A	325.8	324.7	327.7
	S-B	331.6	315.2	328.4
	S-C	327.6	320.5	322.6
	S-0	341.2	339.3	345.3
Full irrigation	F-0	375.4	380.1	382.9

L, M, and S are low, moderate, and severe water deficit, respectively; A, B, and C are Japanese film, Dupont film, and Chinese film, respectively.

## 4 Discussion

### 4.1 Effects of water deficit on appearance quality of citrus fruit under plastic film mulching

The reflectance of films A and B were significantly different from that without mulching, which was consistent with the results of Si et al. (2024). Two types of film increased the light in the middle and lower parts of the canopy, promoted the photosynthesis intensity of leaves, and improved the fruit coloring degree (Zhao et al., 2022; Pacheco et al., 2021). The present study also showed that films A and B significantly increased the appearance quality of citrus, such as fruit coloring degree, single fruit weight, and excellent fruit percentage, and reduced the pericarp thickness. These findings were consistent with the research results of Wang et al. (2022), because the light-supplementing effect of film mulching on the surface makes the fruit uniformly colored and promotes the accumulation of dry matter in the fruit (Lee et al., 2021), thereby increasing the single fruit weight and excellent fruit percentage. The rain-shelter effect of film mulching could effectively slow down the division and growth of the middle cortex cells of pericarp from causing the pericarp to thicken. Wang et al. (2010) also found that reflective plastic film could effectively reduce the pericarp thickness of citrus unshiu.

Moderate water deficit could effectively improve the appearance quality of fruit (Abou Ali et al., 2024). The present study found that water deficit at stage II had no significant effect on coloring degree and pericarp thickness, which were consistent with the study of Zhong et al. (2019), because the fruit began to color at stage IV, so water deficit at stage II did not affect fruit coloring degree. Citrus pericarp thickness (middle cortex) was determined by the anticlinal division and periclinal division between cells, and it grows rapidly at stage III. Therefore, the key period for controlling pericarp thickness was stage III, and water deficit at stage II had slight effect on citrus pericarp thickness (Yu et al., 2021). Ansari et al. (2018) found that moderate water deficit at stage II could increase the single fruit weight and excellent fruit percentage of melon, similar to the results of the present study. On the one hand, moderate water deficit at stage II could effectively inhibit the excessive growth of fruit trees, thus supplying more photosynthetic products to fruit growth and development (Zhang et al., 2018). On the other hand, fruit trees after moderate water deficit could produce transcendence compensation effect, where the photosynthetic rate of trees that have undergone water deficit exercise increases substantially after the restoration of water supply (Kou et al., 2014).

### 4.2 Effects of water deficit on the internal quality of citrus fruit under plastic film mulching

This study found that films A and B could significantly improve the internal quality of fruit soluble reducing sugar, soluble solids, and vitamin C content, similar to the results of Jiang et al. (2014) and Suo et al. (2019). At present, there was a consensus on the sugar-enhancing effect of film mulching, but its effect on organic acids in fruit was still widely divergent, with most studies showing that film mulching effectively reduces organic acid content (Shi et al., 2011; Pacheco et al., 2021), and others showing that film mulching significantly increases it (Jiang et al., 2014). Shi et al. (2011) found that mulching with moisture permeable reflective film significantly reduced the organic acid content of citrus, similar to the findings of the present study. The moisture permeable reflective film is a new type of agricultural mulch made from spunbond PE. It has reflective, rainproof, and breathable properties, and when burned, it produces carbon dioxide and water, resulting in minimal environmental pollution (Shi et al., 2011). The reason could be analyzed from two aspects. First, film mulching decreases the organic acid content of fruit by increasing the fruit water content (Jin et al., 2018). However, this study found that film mulching has slight effect on the juice yield (fruit water content). Second, film mulching decreased the synthesis of titratable acid or increased the catabolism, and finally decreased the organic acid content of fruit (Pacheco et al., 2021). However, Jiang et al. (2014) showed that mulching with silver black double-color film slowed down the utilization of citric acid by reducing the activity of citrate dehydrogenase, thereby increasing the content of organic acids in fruits. The above differences may be related to film mulching materials, film mulching period, and fruit tree varieties. Silver black double-color film is an airtight plastic film that is detrimental to root respiration and microbial growth in soil, potentially leading to poor tree growth. Furthermore, this thin film is prone to damage, allowing rainwater to penetrate into the soil and compromising the effectiveness of water control (Jiang et al., 2014).

This study showed that with the increase in degree of water deficit at stage II, the fruit juice yield decreased significantly, and the soluble solids and reducing sugar content increased significantly, similar to the results of Saitta et al. (2021). Water deficit could limit the expansion and division of pulp cells, reducing the juice sac “storage capacity” to reduce fruit water content (Jiang et al., 2014; Ma et al., 2022). The present study found that the changes in sugar and acid in citrus fruit under water deficit conditions were not synchronized over time (**Table 4**), which could be confirmed that the increase of sugar content in fruits caused by water deficit was not the result of passive water loss. The reason may be that water deficit induces osmotic adjustment mechanism of fruit trees to cope with drought stress (Wang et al., 2019), thus making citrus fruit sugar accumulation increased.

### 4.3 Effects of water deficit on yield and WUE of citrus under plastic film mulching

This study found that the water-saving effect of film C was better than A and B (**Figure 6**), because film C is an impermeable and air-tight, whereas films A and B are rainproof (rainwater cannot pass through the film) and moisture permeable (water and gas in the soil can pass through the film into the air), so the ability of film C to hinder soil moisture evaporation was stronger than that of films A and B. Most studies have shown that water deficits could improve fruit quality without reducing yield or with minimal yield reduction (Perez-Pastor et al., 2014), as confirmed by the results of the present study, which found that low and moderate water deficits at stage II significantly increased citrus yield. However, some studies have shown that water deficit significantly reduces fruit yield (Tong et al., 2022; Cui et al., 2008), and our results also confirmed that severe water deficits at stage II significantly reduced citrus yield. Thus, there was a consensus on regulated deficit irrigation could improve fruit quality, but its effect on yield was still widely divergent. The reasons for the divergence may be related to the period of water deficit, the degree of deficit, the fruit trees age and fertilizer. Different rootstocks demonstrate varying sensitivities to water deficit, attributable to their unique genetic traits and physiological mechanisms, which result in differential growth inhibition (Li et al., 2023). *Fructus aurantii* exhibits strong drought resistance, sustaining growth under water deficient conditions and rapidly adjusting its physiological state to minimize water loss (Rasool et al., 2020). Furthermore, *Fructus aurantii* serves as an excellent rootstock for resistance to citrus tristeza virus (Ghimire et al., 2023); therefore, this study exclusively focuses on *Fructus aurantii* as the rootstock. Future research should compare diverse rootstocks under similar mulching and water deficit conditions to enhance the understanding of their responses.

## 5 Conclusions

Water deficit and film mulching could significantly improve the quality of citrus, M-A and M-B treatments at stage II were the better treatments to improve the quality of citrus. In addition, water deficit and film mulching significantly affected citrus yield, ET, and WUE, among which low and moderate deficit significantly increased yield, whereas severe deficit significantly decreased yield. Film mulching could effectively reduce the effect of severe water deficit on citrus yield reduction. The interaction between water deficit and film mulching also had significant effects on WUE, and the WUE of M-A and M-B treatments was the highest, which was greater than 7.00 kg m^-3^. Therefore, the better treatment should be to control the soil moisture at 70% *θ_f_*–80% *θ_f_* during the young fruit period of citrus under the mulching of films A and B and the quality, yield, and WUE of citrus reached a high level.

## Data Availability

The original contributions presented in the study are included in the article/supplementary material. Further inquiries can be directed to the corresponding authors.
